# The 2013 American College of Rheumatology/European League Against Rheumatism Classification Criteria for Systemic Sclerosis Could Classify Systemic Sclerosis Patients at Earlier Stage: Data from a Chinese EUSTAR Center

**DOI:** 10.1371/journal.pone.0166629

**Published:** 2016-11-16

**Authors:** Dong Xu, Yong Hou, Yuanfang Zheng, Yue Zheng, Mengtao Li, Xiaofeng Zeng

**Affiliations:** Department of Rheumatology and Clinical Immunology, Peking Union Medical College Hospital, Peking Union Medical College and Chinese Academy of Medical Sciences, Key Laboratory of Rheumatology and Clinical Immunology, Ministry of Education, Beijing, 100730, China; Medical University of South Carolina, UNITED STATES

## Abstract

**Objectives:**

To evaluate the performance of the 2013 ACR/EULAR classification criteria for systemic sclerosis (SSc) in clinical practice in a Chinese patient cohort, and to compare outcomes with the 1980 ACR criteria.

**Methods:**

Patients clinically diagnosed with SSc between September 2013 and May 2015 were prospectively recruited from the EUSTAR database of the Peking Union Medical College Hospital. Diagnosis of SSc was based on the evaluation of three experienced rheumatologists. Patients diagnosed with other connective tissue diseases were recruited as disease controls. The 1980 ACR and 2013 ACR/EULAR criteria were applied to the cohort, and patients who fulfilled the criteria were classified as definite SSc patients. Sensitivity and specificity were analyzed for the 2013 and 1980 criteria.

**Results:**

A total of 143 SSc patients and 87 patients with other connective diseases were recruited. 41 (28.7%) and 102 (71.3%) cases were diffuse cutaneous SSc and limited cutaneous SSc, respectively. Although the sensitivity of the 2013 criteria (94.4%) exceeded the 1980 criteria (72.7%) (P<0.001), the 1980 and 2013 criteria sets showed no significant difference in specificity (97.7% and 93.1%, respectively, P = 0.278). The sensitivity of the 2013 criteria was significantly higher than the 1980 criteria in some SSc subgroups (e.g., lcSSc, abnormal pattern of nailfold videocapillaroscopy [NVC] and presence of Raynaud’s phenomenon [RP]) compared to others.

**Conclusions:**

Relative to the 1980 ACR criteria, in Chinese SSc patients the new 2013 ACR/EULAR criteria had similar specificity and higher sensitivity, especially for patients with mild skin thickening or prominent microvascular diseases.

## Introduction

Systemic sclerosis (SSc) is an autoimmune disease characterized by fibrosis, microvascular disease, and presence of autoantibodies. In China, two fatal manifestations, interstitial lung disease (ILD) and pulmonary arterial hypertension (PAH), are the most common SSc complications among connective tissue diseases [1,2]. Thus, classification of SSc during the early disease stages is critical so that treatment can begin sooner. The first widely accepted classification criteria for SSc were published in 1980, and the high specificity of 1980 criteria helped minimize false-positive diagnoses [3]. Nonetheless, improved classification criteria that could diagnose SSc even earlier were urgently needed. In 2001 LeRoy and Medsger proposed modifications to the criteria used to diagnose early SSc [4]. Recently, a joint committee of the American College of Rheumatology (ACR) and the European League Against Rheumatism (EULAR) formulated a new set of SSc classification criteria, which has proven to have increased sensitivity over the 1980 criteria while still preserving the specificity determined from studies conducted in other countries [5,6]. The objective of this study was to validate the sensitivity and specificity of the new classification criteria in Chinese SSc patients.

## Patients and Methods

### Patients

Patients who were clinically diagnosed with SSc between September 2013 and May 2015 were prospectively recruited from the EUSTAR database of the Peking Union Medical College Hospital (PUMCH). Diagnosis of SSc was based on the evaluation of three experienced rheumatologists from PUMCH. SSc patients were not required to meet any formal classification criteria. Patients diagnosed with other connective tissue diseases were recruited as disease controls according to the respective classification criteria.

Pulmonary arterial hypertension (PAH) was defined as a mean pulmonary arterial pressure of > 25 mmHg, together with a pulmonary capillary wedge pressure of < 15 mmHg determined by right heart catheterization(RHC). Pulmonary fibrosis was defined as ground glass opacification or fibrosis on high-resolution computed tomography (HRCT).

### Methods

This study was approved by the Medical Ethics Committee of Peking Union Medical College Hospital and all subjects provided informed written consent. Demographic features, clinical characteristics (including Raynaud’s phenomenon, puffy fingers, telangiectasias, and digital pitting), and laboratory findings (anti-centromere, anti-Scl-70, and anti-RNA polymerase III antibodies, echocardiography and HRCT, and nailfold videocapillaroscopy (NVC) test) were collected for all patients. Patients were classified into limited and diffuse cutaneous subsets based on the definition of Leroy *et al*. [4]. Disease duration was defined as the time from the first non-RP SSc manifestation to entry. The 1980 ACR and 2013 ACR/EULAR criteria were applied to evaluate the patients diagnosed with SSc clinically and disease controls; patients who fulfilled each criterion were classified as definite SSc patients. Sensitivity and specificity were evaluated for each set of classification criteria. Sensitivity for various SSc subgroups was also evaluated.

### Statistical analysis

The Statistical Package for the Social Sciences (SPSS) version 17.0 (SPSS, Chicago, IL) was used for data processing and analysis. Quantitative results were presented as mean ± standard deviation (normal distribution), or median, interquartile range (non- normal distribution). The x2 test was used to analyze enumeration data. P < 0.05 was considered to be statistically significant.

## Results

A total of 143 SSc patients and 87 patients with other connective diseases were recruited for this study (Table 1). All patients were of Han nationality. Of the 143 SSc patients, 41 (28.7%) and 102 (71.3%) cases were diffuse cutaneous SSc (dcSSc) and limited cutaneous SSc (lcSSc), respectively. Disease duration was 63.15 ± 59.32 months. 137 of 143 patients with SSc had RP, and it was the initial symptom in 113 patients. The duration from RP and first symptom to entry were 86.61±78.22 months and 87.18±77.22 months, respectively. The clinical features of SSc patients and disease controls are listed in Table 2. On the other hand, pulmonary artery systolic pressure > 40 mmHg at rest based on an echocardiogram test was present in 33 patients in 143 SSc patients. 22 in 33 patients had done the RHC and were confirmed to have PAH. Though the other 11 patients didn’t do the RHC, no matter whether PAH was present in these patients, the validation results for 1980 and 2013 criteria were same. Rodnan scores were 5.0 ± 5.6 in SSc patients. The other clinical features were as following: joint involvement 41.3% (59/143), myositis 10.5% (15/143), gastrointestinal involvement 59.4% (85/143), gastroesophageal reflux 49.7% (71/143), heart conduction block 2.8% (4/143), and scleroderma crisis 0%.

**Table 1:** Disease spectrum of other connective tissue diseases used as controls.

	Cases (%)	RP Cases
SLE	45 (51.7)	32
Polymyositis /Dermatomyositis	14 (16.1)	2
MCTD	9 (10.3)	9
UCTD	6 (6.9)	5
Systemic vasculitis	5 (5.7)	1
Primary Sjögren’s syndrome	5 (5.7%)	2
Fasciitis	2(2.3%)	0
Localized scleroderma	1(1.1%)	0
Total	87	51 (58.6%)

SLE, systemic lupus erythematosus; MCTD, Mixed connective tissue disease; UCTD, undifferentiated connective tissue disease; RP,Raynaud’s phenomenon.

**Table 2:** The demagraphic and clinical features of SSc patients and disease controls.

	SSc patients (n = 143)	Disease controls (n = 87)	P value
Age (years)	46.92 ± 11.74	38.72 ± 11.91	<0.001
Gender (F/M)	133/10	73/14	0.029
RP	137 (95.8%)	52 (59.8%)	<0.001
Puffy fingers	68 (47.6%)	18 (20.7%)	<0.001
Sclerodactyly	111 (77.6%)	2 (2.3%)	<0.001
Digital ulcers	37 (25.9%)	4 (4.6%)	<0.001
Pitting scars	21 (14.7%)	2 (2.3%)	0.002
Telangiectasias	52 (36.4%)	2 (2.3%)	<0.001
PAH	22/132 (16.7%)	13 (14.9%)	0.135
ILD	104 (72.7%)	25 (28.7%)	<0.001
ACA	23 (16.1%)	6 (6.9%)	0.042
Anti-Scl70 antibody	57 (39.3%)	3 (3.4%)	<0.001
Anti-polymerase III antibody	0(0%)	0(0%)	1.000

RP, Raynaud’s phenomenon; PAH, pulmonary arterial hypertension; ILD, interstitial lung disease; ACA, anticentromere antibody.

Of the 143 SSc and 87 other disease patients, 104 and 2 patients, respectively, satisfied the 1980 criteria. On the other hand, 135 of the 143 SSc patients and 6 of 87 patients in the other connective diseases group satisfied the 2013 criteria. The sensitivity of the 2013 classification criteria for SSc (94.4%) exceeded the 1980 classification criteria (72.7%) (P<0.001). However, there was no significant difference between the specificity of the two classification criteria (93.1% and 97.7% for 2013 and 1980, respectively; P = 0.278).

Further, we did a sensitivity analysis excluding patients with MCTD since these patients frequently had prominent SSc features and often evolved into SSc, The results were similar. Of the 143 SSc and 78 other disease patients, 104 and 2 patients, respectively, satisfied the 1980 criteria. On the other hand, 135 of the 143 SSc patients and 5 of 78 patients in the other connective diseases group satisfied the 2013 criteria. The sensitivity of the 2013 classification criteria for SSc 94.4% exceeded the 1980 classification criteria 72.7% (P<0.001).

The sensitivities of the 1980 ACR and 2013 ACR/EULAR in various subgroups of SSc patients were further evaluated. There were no significant differences between 1980 and 2013 criteria in male (90% vs. 90%), dsSSc (97.6% vs. 100%), presence of digital ulcers (86.5% vs. 100%), presence of pitting scars (85.7% vs. 100%), absence of abnormal NVC patterns (86.4% vs. 86.4%), absence of RP (50% vs. 66.7%), anti-Scl70 antibody positivity (96.5% vs. 100%). On the other hand, there were significant increased sensitivity in 2013 than 1980 criteria in female (71.4% vs. 94.7%), lcSSc (62.7% vs. 92.2%), absence of digital ulcers (67.9% vs. 92.5%) or pitting scars(70.5% vs. 93.4%), presence of abnormal NVC patterns (70.2% vs. 95.9%) or RP (73.7% vs. 95.6%), anti-Scl70 antibody negativity(57% vs. 90.7%). Moreover, there were also significant increased sensitivity in 2013 than 1980 criteria in disease duration < 3 or ≥ 3 years, presence or absence of Sclerodactyly, telangiectasia, PAH, ILD, ACA and anti-RNP antibody.

## Discussion

Skin thickening is a characteristic manifestation of SSc, and many physicians recognize this disease by this feature. However, the diagnosis of SSc patients with mild skin thickening always was delayed. That was a deficiency for the 1980 ACR SSc classification criteria. Consistent with previous studies that examined disease characteristics in patients of other nationalities, our study also showed that the 2013 ACR/EULAR SSc classification criteria had improved sensitivity without affecting specificity in Chinese Han populations [5,6,7–10] (Table 3). Also, the 2013 ACR/EULAR criteria had significantly increased sensitivity relative to the 1980 criteria in most SSC patient subgroups, with the exception of patients with more extensive skin thickening, e.g., diffuse cutaneous SSc. These patients had a high rate of diagnosis even using 1980 criteria due to extensive skin involvement. The 2013 ACR/EULAR criteria had higher sensitivity in patients with mild skin thickening. Interestingly, in our study we found that the 2013 criteria did not produce increased sensitivity in either male patients or patients who were positive for anti-Scl-70 antibody, which may be due to the greater frequency of dcSSc patients in these two subgroups (60% and 53%, respectively).

**Table 3:** Sensitivity and specificity of 1980 ACR and 2013 ACR/EULAR criteria in different countries.

	Patient numbers		1980 ACR		2013 ACR/EULAR	
	SSc	Control	Sensitivity	Specificity	Sensitivity	Specificity
ACR/EULAR (13 centers in North America, 10 centers in Europe)						
Derivation sample [5]	100	100	81	77	95	93
Validation sample [5]	268	137	75	72	91	92
Turkey (3 centers) [6]	131	103	85.3	100	94.4	98.1
Canada (15 centers) [7]	724		88.3		98.3	
Norway (single center) [8]	391/425*	178	75		96	90
Switzerland (single center) [9]	304		53.5		79.6	
Spain(single center)[10]	106	221	76.4	96.5	98.1%	94.6%
Our study (single center)	143	87	72.7	97.7	94.4	93.1

*Because prescleroderma and sine SSc, by definition, would not be tested by the 1980 ACR criteria, only 391 patients was classifiable by 1980 criteria

On the other hand, NVC is widely used to differentiate secondary RP from primary RP [11]. The 2013 criteria includes the NVC test, and our study showed the sensitivity of the 2013 criteria was significantly increased relative to the 1980 criteria in patients with an abnormal NVC pattern, but there was no difference between the old and new criteria for patients with a normal NVC pattern. As we know, abnormal NVC pattern could indicate and accompany with the presence of other microvascular diseases, for example Raynaud’s phenomenon, PAH, and telangiectasia., and these manifestations of microvascular diseases were all included in the 2013 criteria, but not in the 1980 criteria. This maybe was the reason for increased sensitivity in patients with an abnormal NVC pattern. Similarly, one study from a capillaroscopy clinic showed that one of individual variables in 2013 criteria with the best sensitivity was capillaroscopic abnormalities(81.1%)[10]. SSc patients with prominent microvascular diseases maybe could be classified earlier with the 2013 criteria.

To date, only four studies have evaluated the specificity of the old and/or new criteria. As with previous studies, our study showed that the specificity of the 2013 and 1980 criteria was high and the two criteria sets exhibited no significant differences. However, the 1980 criteria had higher specificity than that determined by the ACR/EULAR group, which is similar to results obtained in studies performed in Turkey and Spain. These differences could be due to the choice of controls. The characteristics of the controls in the Turkey study and our study were similar, and almost all control patients had connective tissue diseases with ANA positivity. However, the ACR/EULAR study included more cases with non-CTD scleroderma-like diseases, which had skin thickening similar to that seen in SSc. The inclusion of these patients may thus reduce the specificity of the 1980 criteria in the ACR/EULAR study.

This is the first study to evaluate the performance of the new 2013 criteria in a Han Chinese population. In Chinese SSc patients, the new 2013 criteria had higher sensitivity and similar specificity compared with the 1980 criteria, especially in patients with mild skin thickening or prominent microvascular diseases. It could clarify the SSc patients at earlier stage.

## Supporting Information

S1 Data Primiary data of patients with systemic sclerosis. This is the data about the patients with SSc.
(XLS)

S2 Data Primiary data of patients with disease control. This is the data about the patients with disease control.
(XLS)
